# Bibliographical Notices

**Published:** 1844-10-19

**Authors:** 


### BIBLIOGRAPHICAL NOTICES.

*Cyclopædia of Practical Medicine.* Parts XIII, XIV, XV.

Some time since, we noticed the conclusion of the second volume, or No. XII. of this valuable work. Since then we have received three more numbers, lacking nothing of the excellence of those which preceded. From the rapid and regular manner in which the publication proceeds, it is evident that the whole will be completed within the time promised by the enterprising publishers.

*An Essay on the Philosophy of Medical Science.* By Elisha Bartlett, M. D., Professor of the Theory and Practice of Medicine, in the University of Maryland: 8vo. pp. 312. Philadelphia, Lea & Blanchard, 1844.

Considering the great respectability of the source from whence it comes, this is a remarkable book for the present day. It is nothing less than an attempt to revive the doctrines of *Empiricism*, as taught in the earliest ages, to the utter exclusion of all "*rationalism*," or reasoning in medicine. With the author, practical medicine is an "*empirical art.*" All our knowledge is confined to direct observation; observation must guide us in all cases; and where that fails, we have no light, no guide whatever. That we may not do either him or ourselves injustice, we will quote his own language.

"Writers upon the science and art of medicine, have always been, so far as the subject now before us is concerned, divided into two classes, or schools; those of the *rationalists*, and of the *empirics*. The former have always been, and still continue to be, the most numerous and powerful. Their doctrines have pervaded and governed the medical world. They claim to be more *philosophical* than their opponents, the *empirics*. They profess to be governed and guided in their theory and practice, by what they are pleased to call *rational principles*. They allege that their therapeutics is founded upon rational *indications*. They claim, not merely to cure diseases, but cure to them *philosophically*, and in conformity to their *rational principles*. They claim, not merely to have ascertained the relationship which exists between diseases and their remedies, but to understand the *nature* and the *reasons* of this relationship. They pretend to explain the *mode* and *manner* in which their remedies produce their results. Their doctrine is, that therapeutics is *founded upon* pathology; that the former is deduced from the latter. They are very confident in their knowledge of the intimate *modus operandi* of their remedies. The empirics, on the other hand, deny all this. They say, that our knowledge of the relationship between diseases and their modifiers, is the sole and exclusive result of observation of this relationship itself. They disclaim any knowledge of the intimate and essential nature of this relationship. They deny that any acquaintance, however complete and accurate, with the phenomena of pathology, could ever of itself have led to a knowledge of the relations, which exist between these phenomena, and those substances and influences in nature, endowed with the property of arresting or controlling these phenomena. They deny, that therapeutics is founded upon pathology. They deny, that by any process of reasoning, the former can be deduced from the latter. This doctrine, I hardly need say, is the doctrine of this essay," &c., (pages 103, 4, 5.)

Professor Bartlett maintains that our knowledge of physiology is not deducible from our knowledge of anatomy," that "our knowledge of pathology is not deducible from our knowledge of physiology," and that "therapeutics is not founded upon pathology." Now we think it would be somewhat difficult for him to point out, consistently with these views, any advantage to be derived, in the treatment of diseases, from the study of these subjects; but we can hardly think that he is yet prepared to discard them from his course of medical instruction.

We cannot, however, see how he can justify spending so much time as is necessary to acquire even a tolerable knowledge of anatomy, physiology, pathology, and therapeutics, when he declares emphatically, that "*Therapeutics is not founded upon pathology. The former cannot be deduced from the latter. It rests wholly upon experience. It is, absolutely and exclusively, an empirical art.*"—(pages 113–14.)

Our author seems to be greatly enamoured of the French school, and especially of the numerical method of Louis, to whom, indeed, he has dedicated his volume. He obviously does not subscribe to the saying that "there are more false facts than false theories:" yet, the frequency with which results of experiments, called observations, are set down as facts, which turn out not to be so, is well calculated to render the mind skeptical, and especially when these so-called facts are not consonant with general observation. A striking example of this, and of the readiness with which the mind embraces as facts what it is already predisposed to believe, is exhibited in the work before us. Speaking of the abandonment of the use of opium to procure sleep, in delirium tremens, the author observes: "The therapeutical reform, thus commenced, has been recently completed by the remarkable and unparalleled success which has followed the alcoholic treatment of the disease by Dr. Gerhard." This, we have no doubt, refers to the practice of Dr. Gerhard in the Philadelphia Hospital.

In the last number of the *American Journal of the Medical Sciences*, (October, 1844,) there is a statement of the "Statistics of the Causes of Death" in that Institution "during a period of twelve years," by Dr. Tabb, lately one of the resident medical officers. By that statement it appears that no deaths occurred of the large number admitted into the hospital, except among those in the third stage. Of those treated by Dr. Gerhard, (with alcohol,) *two* were admitted in the third stage, one of whom was *cured*, and one *died*. Of those treated by Dr. Dunglison (without "a drop of alcohol") *ten* were admitted in the third stage, of whom *one* died. So that the "unparalleled success" which "completed" the reform by substituting *alcohol* for *opium*, under which fifty per cent. died of those in the third stage, has been surpassed, and the reform further "completed" by abandoning alcohol, whereby only *one-tenth* died of those admitted in the third stage. Thus we see that whether our observations are "complete" or not, depends very much upon the condition of the mind by which they are to be judged—the mind will be easily satisfied if they confirm what *in theory* it has already decided.

A great and pervading error in the present work, it appears to us, is in regarding the living body in the light of a mere physical machine; or, at least, in not sufficiently considering the vital reactions that are continually occurring, under the influence of mental and physical causes.

There are some other points which we should be disposed to object to, but that our limits confine us to "*notices*," and do not allow of extended analysis or criticism, however interesting or worthy the publication:—a few words, therefore, must close what we have to say.

The leading doctrine of this work, as we have remarked, is many centuries old—has been dead and buried, in fact, some two thousand years, and is now again brought forth, stripped of its cerements, dressed up the rich garments afforded by modern literature, and the breath of life attempted to be breathed into its nostrils—with what success, remains to be seen. Never before, certainly, have we seen the same doctrine put forth with more confidence, or defended with more ability, and in a style so well calculated to captivate the understanding.

Nevertheless, so little are the views advanced in accordance with the opinions of the present generation of men, that most people would deem any attempt at their refutation a bootless task, In dragging forth, at the present time, doctrines so long entombed, it seems to us that the able author has placed himself very much in the predicament of one who should deny the sanity of all around him. The judges in the case are much more likely to decide against him than against themselves.

